# Pregnancy associated cancer, timing of birth and clinical decision making—a NSW data linkage study

**DOI:** 10.1186/s12884-023-05359-1

**Published:** 2023-02-09

**Authors:** Nadom Safi, Zhuoyang Li, Antoinette Anazodo, Marc Remond, Andrew Hayen, David Currow, David Roder, Nada Hamad, Michael Nicholl, Adrienne Gordon, Jane Frawley, Penelope Fotheringham, Elizabeth Sullivan

**Affiliations:** 1 College of Health, Medicine and Wellbeing, University of Newcastle, 130 University Drive, Callaghan, NSW 2308 Australia; 2 Hunter Medical Research Institute, New Lambton Heights, NSW 2305 Australia; 3 Prince of Wales Hospital, Nelune Comprehensive Cancer Centre, Randwick, NSW 2031 Australia; 4 School of Public Health, University of Technology Sydney, Ultimo, NSW 2007 Australia; 5 University of Wollongong, the Vice-Chancellor’s Unit, NSW, Wollongong, 2522 Australia; 6 University of South Australia, Population Health, Beat Cancer Project, Adelaide, SA Australia; 7 The Kinghorn Cancer Centre, Darlinghurst, NSW 2010 Australia; 8 Faculty of Medicine and Health, The University of Sydney, Camperdown, NSW Australia

**Keywords:** Pregnancy, Neoplasms, Incidence, Pregnancy outcome, Perinatal death

## Abstract

**Background:**

The incidence of pregnancy-associated cancer (PAC), comprising cancer diagnosed during pregnancy or within one year postpartum, is increasing. We investigated the obstetric management and outcomes of women with PAC and their babies.

**Methods:**

A population-based observational study of all women who gave birth between 1994 and 2013 in New South Wales, Australia. Women were stratified into three groups: those diagnosed during pregnancy (gestational cancer group), those diagnosed within one year of giving birth (postpartum cancer group), and a no-PAC group. Generalized estimating equations were used to examine the association between PAC and adverse maternal and neonatal outcomes.

**Results:**

One million seven hundred eighty-eight thousand four hundred fifty-onepregnancies were included—601 women (614 babies) were in the gestational cancer group, 1772 women (1816 babies) in the postpartum cancer group, and 1,786,078 women (1,813,292 babies) in the no-PAC group. The overall crude incidence of PAC was 132.7/100,000 women giving birth. The incidence of PAC increased significantly over the twenty-year study period from 93.5/100,000 in 1994 to 162.5/100,000 in 2013 (2.7% increase per year, 95% CI 1.9 – 3.4%, *p*-value < 0.001). This increase was independent of maternal age. The odds of serious maternal complications (such as acute abdomen, acute renal failure, and hysterectomy) were significantly higher in the gestational cancer group (adjusted odds ratio (AOR) 5.07, 95% CI 3.72 – 6.90) and the postpartum cancer group (AOR 1.55, 95% CI 1.16 – 2.09). There was no increased risk of perinatal mortality in babies born to women with PAC. However, babies of women with gestational cancer (AOR 8.96, 95% CI 6.96 – 11.53) or postpartum cancer (AOR 1.36, 95% CI 1.05 – 1.81) were more likely to be planned preterm birth. Furthermore, babies of women with gestational cancer had increased odds of a severe neonatal adverse outcome (AOR 3.13, 95% CI 2.52 – 4.35).

**Conclusion:**

Women with PAC are more likely to have serious maternal complications. While their babies are not at increased risk of perinatal mortality, they are more likely to experience poorer perinatal outcomes associated with preterm birth. The higher rate of birth intervention among women with gestational cancers reflects the complexity of clinical decision-making in this context.

**Supplementary Information:**

The online version contains supplementary material available at 10.1186/s12884-023-05359-1.

## Background

Cancer diagnosed during pregnancy or within one year of giving birth is referred to as pregnancy-associated cancer (PAC) [1–3]. PAC incidence has increased over recent decades, and this may be partially explained by a global trend of increasing maternal age [2]. Although the diagnosis of PAC is relatively rare, with estimates of incidence varying from 71 to 172 cases per 100,000 women giving birth, [4] it is associated with adverse outcomes for affected mothers and their babies. Adverse maternal outcomes include higher rates of hysterectomy, blood transfusion, thromboembolism, sepsis, delivery by cesarean section, and preterm birth [2, 3, 5–9]. Adverse neonatal outcomes include elevated risk of lower birthweight, smaller for gestational age, lower Apgar scores, and greater need for resuscitation [2, 3, 5–9]. Cancer management during pregnancy is particularly challenging as treating clinicians must carefully appraise the likely benefits of any intervention to the mother against potential harm to her fetus [2, 10, 11].

Women with postpartum cancer are included under the definition of PAC as the pathogenic origin of their cancer is likely to have been present during pregnancy [8]. Nonetheless, as women diagnosed with postpartum cancer generally receive obstetric management similar to the general population, any differences in maternal and perinatal outcomes between these women and those diagnosed during pregnancy are likely to reflect differences in management rather than impacts of cancer itself.

Contemporary data on PAC in Australia are limited [2]. The aim of this study was to describe the incidence, birth management and maternal outcomes of women with PAC, and the perinatal outcomes of their babies, in New South Wales (NSW) to inform the evidence base and clinical decision-making.

## Methods

### Study design and population

We conducted a population-based cohort study using data from NSW, Australia. The study population comprised all women who gave birth between 1 January 1994 and 31 December 2013 and their babies. Birth was defined as all live births, and stillbirths of at least 400 g birth weight or at least 20 weeks’ gestation [12]. For women with more than one birth during the study, each pregnancy that met the inclusion criteria was included as a separate event.

Included women and their babies were stratified as follows: the “gestational group” comprised women diagnosed with invasive cancer during pregnancy and their babies; the “postpartum group” comprised women diagnosed with invasive cancer within one year of giving birth and their babies; the comparison “no-PAC group” comprised all other women who were not diagnosed with PAC and their babies. We utilized two datasets to identify and classify the cohort. The Perinatal Data Collection was used to identify women who gave birth during the study period while the New South Wales (NSW) Cancer Registry was used to identify which of these women had a diagnosis of cancer during pregnancy or within one year of giving birth [13] Details regarding ICD10_AM codes to identify women with cancer and classification to specific cancer groups are available from the NSW Cancer Registry data dictionary [14]. Note that, women were included in the gestational group or the postpartum group only if their first reported diagnosis of invasive cancer was during pregnancy or within one year of giving birth. Stage of cancer was determined from the NSW Cancer Registry [14].

### Data sources

Data from eight NSW administrative population datasets were analyzed (Supplemental Table 1). NSW Centre for Health Record Linkage [15] performed probabilistic data linkage to identify and merge records relating to the same person from separate datasets. Each person was assigned a unique project person number to maintain privacy.

### Study outcomes

Maternal outcomes included maternal morbidities, pregnancy and birth management outcomes (including labor induction and birth by cesarean section (CS)), maternal death and discharge status. A composite outcome measure (severe maternal morbidity outcome indicator (MMOI)) was assessed using a validated list of fourteen ICD10 diagnosis codes and eleven Australian Classification of Health Interventions (ACHI) ICD10 procedure codes developed by Roberts and colleagues (Supplemental Table 2) [16]. Any woman admitted to hospital during pregnancy, or within 42 days postpartum, with any of these diagnoses or procedures was deemed to have had a severe maternal morbidity outcome.

Neonatal outcomes included: perinatal death (stillbirth ≥ 20 weeks, liveborn neonatal death < 28 days), preterm birth (< 37 weeks), induced labor or cesarean section (CS) without labor where the main indication for CS was not fetal distress (this is a proxy measure of planned birth), low birthweight (< 2500 g), small-for-gestational-age (SGA), [17, 18] low 5-min Apgar score (< 7), admission to intensive care (neonatal intensive care unit (NICU) or special care nursery (SCN)), prolonged hospital stay (≥ 5 days), and congenital malformation. A composite outcome measure (severe neonatal adverse outcome indicator (NAOI)) was assessed using a validated list of twelve ICD10 diagnosis codes and seven ACHI ICD10 procedure codes developed by Lain and colleagues (Supplemental Table 3) [19]. Any baby with a relevant diagnosis or procedure code recorded in their birth record or in any hospital transfer admission before the first discharge home was deemed to have had a severe neonatal adverse outcome.

### Data analysis

Results for continuous measures are presented as mean and standard deviation while results for nominal data are presented as counts and percentages. When comparing study groups, one-way ANOVA was used to assess differences in continuous variables, while a Chi-squared test was used to assess differences in categorical variables.

We used Poisson regression models to examine the estimated increase in PAC incidence over the study period, unadjusted and adjusted for women’s age in each group. We estimated the direct age-standardized incidence rate using 1994 Australian population data for women giving birth as the standard population.

Generalized estimating equation (GEE) models with exchangeable correlation structure were used to compare the likelihood of adverse maternal and neonatal outcomes between study groups. Covariates available for analysis were: maternal age, country of birth, remoteness, parity, timing of antenatal care, smoking during pregnancy, plurality, pre-existing morbidities, a CS in a previous pregnancy, and place of birth (public or private). Variables with a *p*-value of < 0.25 in univariate analyses, and biologically plausible factors identified in the literature as predictive of selected outcomes, were entered into multivariable models. Final models were determined by considering collinearity, statistical significance, and goodness-of-fit. Results from these models are presented as odds ratios (OR) and adjusted odds ratios (AOR) with 95% confidence intervals (CI).

Findings with a *p*-value < 0.05, or a CI not including 1, were considered statistically significant. Data were analyzed using R Core Team software (2020) [20] and IBM SPSS Version 27 (IBM Corporation).

## Results

We identified 1,788,451 pregnancies resulting in the birth of 1,815,722 babies. Of these, 601 women (who gave birth to 614 babies) were diagnosed during pregnancy (gestational group) and 1772 (1816 babies) were diagnosed within one year of delivery (postpartum group). The no-PAC group comprised 1,786,078 women who gave birth to 1,813,292 babies.

The overall estimated PAC crude incidence was 132.7/100,000 women giving birth, increasing from 93.5/100,000 in 1994 to 162.5/100,000 in 2013 (2.7% increase per year, 95% CI 1.9 – 3.4%, *p*-value < 0.001). The overall PAC age-standardized incidence was 118.0/100,000.

The overall estimated gestational cancer crude incidence was 33.6/100,000 women giving birth, increasing from 26.6/100,000 in 1994 to 43.0/100,000 in 2013 (3.0% increase per year, 95% CI 1.6 – 4.5%, *p*-value < 0·001). The overall gestational cancer age-standardized incidence was 29.9/100,000.

The overall estimated postpartum cancer crude incidence was 99.1/100,000 women giving birth, increasing from 67.0/100,000 in 1994 to 119.5/100,000 in 2013 (2.5% increase per year, 95% CI 1.7 – 3.4%, *p*-value < 0·001). The overall postpartum cancer age-standardized incidence was 88.1/100,000 (Supplemental Fig. 1).

After adjusting for age in the Poisson regression models, the calculated increases in PAC incidence (1.7% increase per year, 95% CI 1.0 – 2.4%, *p*-value < 0·001), gestational cancer (2.1% increase per year, 95% CI 0.6 – 3.5%, *p*-value < 0·001), and postpartum cancer (1.5% increase per year, 95% CI 0.7 – 2.4%, *p*-value < 0·001) remained statistically significant.

Demographic features of the women included in the analysis are presented in Table 1. There was no significant difference in the mean maternal age of the gestational group (32.3 ± 5.3 years) and the postpartum group (32.4 ± 5.2 years) (*p*-value = 0.948). However, women in both these study groups were significantly older than women in the no-PAC group (29.6 ± 5.6 years; mean difference 2.7 years (95% CI 2.2 – 3.3) and 2.8 years (95% CI 2.5 – 3.1) respectively, *p*-value < 0·001 in both instances). The prevalence of pre-existing diabetes (1.0% vs. 0.6%, *p*-value = 0.003) and pre-existing cardiovascular disease (4.5% vs. 2.6%, *p*-value < 0·001) was significantly greater in the PAC group than in the no-PAC group (Table 1).

**Table 1:** Demographic and baseline features of women giving birth in NSW between 1994 and 2013

Factors	Gestational	Postpartum	no-PAC	Total
	**N(%)**	**N(%)**	**N(%)**	**N(%)**
**Total**	601(100.0)	1772(100.0)	1,786,078 (100.0)	1,788,451(100.0)
**Age group**				
< 30	178(29.6)	489(27.6)	863,125(48.3)	863,792(48.3)
30—34	196(32.6)	656(37.0)	570,119(31.9)	570,971(31.9)
35—39	177(29.5)	487(27.5)	292,292(16.4)	292,956(16.4)
> = 40	50(8.3)	140(7.9)	59,829(3.3)	60,019(3.4)
Unknown	0(0.0)	0(0.0)	713(0.0)	713(0.0)
**Country of birth**				
Australia	444(73.9)	1298(73.3)	1,263,082(70.7)	1,264,824(70.7)
Overseas	157(26.1)	474(26.7)	522,996(29.3)	523,627(29.3)
**Remoteness**				
Major Cities	477(79.4)	1387(78.3)	1,356,994(76.0)	1,358,858(76.0)
Inner Regional	97(16.1)	293(16.5)	305,696(17.1)	306,086(17.1)
Outer Regional	23(3.8)	74(4.2)	91,655(5.1)	91,752(5.1)
Remote or very remote	3(0.5)	14(0.8)	13,262(0.7)	13,279(0.7)
Unknown	1(0.2)	4(0.2)	18,471(1.0)	18,476(1.0)
**Parity**				
0	236(39.3)	603(34.0)	732,969(41.0)	733,808(41.0)
> = 1	365(60.7)	1169(66.0)	1,052,856(58.9)	1,054,390(59.0)
Unknown	0(0.0)	0(0.0)	253(0.0)	253(0.0)
**History of CS**^a^				
Yes	82(15.2)	260(16.6)	194,555(12.5)	194,897(12.5)
No	459(84.8)	1303(83.4)	1,363,503(87.5)	1,365,265(87.5)
**Plurality**				
Singleton	588(97.8)	1729(97.6)	1,759,408(98.5)	1,761,725(98.5)
Multiple birth	13(2.2)	43(2.4)	26,670(1.5)	26,726(1.5)
**Antenatal care**				
< 14 weeks	418(69.6)	1274(71.9)	1,203,102(67.4)	1,204,794(67.4)
14—20 weeks	122(20.3)	329(18.6)	384,915(21.6)	385,366(21.5)
> 20 weeks	52(8.7)	141(8.0)	170,828(9.6)	171,021(9.6)
Unknown	9(1.5)	28(1.6)	27,233(1.5)	27,270(1.5)
**Smoking during pregnancy**				
Yes	52(8.7)	208(11.7)	276,270(15.5)	276,530(15.5)
No	545(90.7)	1563(88.2)	1,504,790(84.3)	1,506,898(84.3)
Unknown	4(0.7)	1(0.1)	5018(0.3)	5023(0.3)
**Place of birth**				
Tertiary hospital	236(39.3)	484(27.3)	490,763(27.5)	491,483(27.5)
Private hospital	153(25.5)	483(27.3)	388,892(21.8)	389,528(21.8)
Public hospital	212(35.3)	805(45.4)	906,420(50.7)	907,437(50.7)
Unknown	0(0.0)	0(0.0)	3(0.0)	3(0.0)
**Pre-existing hypertension**				
Yes	6(1.0)	23(1.3)	15,723(0.9)	15,752(0.9)
No	595(99)	1749(98.7)	1,770,355(99.1)	1,772,699(99.1)
**Pre-existing diabetes**				
Yes	4(0.7)	20(1.1)	10,037(0.6)	10,061(0.6)
No	597(99.3)	1752(98.9)	1,776,041(99.4)	1,778,390(99.4)
**CKD**^bc^				
Yes	2(0.5)	7(0.6)	6402(0.6)	6411(0.6)
No	406(99.5)	1177(99.4)	1,077,957(99.4)	1,079,540(99.4)
**CVD**^bd^				
Yes	14(3.4)	57(4.8)	28,534(2.6)	28,605(2.6)
No	394(96.6)	1127(95.2)	1,055,825(97.4)	1,057,346(97.4)

^a^Data were available from 1998 onwards and denominator was adjusted accordingly

^b^Data were available from Jul 2001 onwards and denominator was adjusted accordingly

^c^Hospital admission due to chronic kidney disease prior to pregnancy

^d^Hospital admission due to cardiovascular disease prior to pregnancy

Table 2 presents the cancer characteristics of women diagnosed with PAC. The mean age at diagnosis in the postpartum group (32.9 ± 5.2 years) was significantly higher than in the gestational group (32.0 ± 5.3 years, *p*-value < 0·001, difference in mean of 0.9 years, 95% CI 0.4, 1.4). Melanoma and breast cancer were the most prevalent cancers diagnosed both during pregnancy and postpartum. Of women in the gestational group, 125 (20.8%) were diagnosed in the first trimester, 234 (38.9%) in the second trimester, and 242 (40.3%) in the third trimester.

**Table 2:** Cancer characteristics of women diagnosed with PAC in NSW between 1994 and 2013

Factors	Gestational	Postpartum	Total
	**N(%)**	**N(%)**	**N(%)**
**Total**	601(100.0)	1772(100.0)	2373(100.0)
**Age at diagnosis**			
< 25	45(7.5)	92(5.2)	137(5.8)
25 – 39	126(21)	296(16.7)	422(17.8)
30 – 34	194(32.3)	616(34.8)	810(34.1)
35 – 39	182(30.3)	560(31.6)	742(31.3)
> = 40	54(9.0)	208(11.7)	262(11.0)
**Cancer group**			
Bone and Connective Tissue	7(1.2)	45(2.5)	52(2.2)
Breast	124(20.6)	400(22.6)	524(22.1)
Colorectal	22(3.7)	101(5.7)	123(5.2)
Eye	3(0.5)	5(0.3)	8(0.3)
Gynecological	70(11.6)	191(10.8)	261(11.0)
Head_Neck	12(2.0)	31(1.7)	43(1.8)
Lymphohematopoietic	77(12.8)	147(8.3)	224(9.4)
Neurological	10(1.7)	46(2.6)	56(2.4)
Other	8(1.3)	16(0.9)	24(1.0)
Respiratory	5(0.8)	22(1.2)	27(1.1)
Skin^a^	187(31.1)	395(22.3)	582(24.5)
Thyroid_Endocrine	65(10.8)	313(17.7)	378(15.9)
Upper GI	6(1.0)	36(2.0)	42(1.8)
Urogenital	5(0.8)	24(1.4)	29(1.2)
**Stage of cancer**			
Localized	296(49.3)	925(52.2)	1221(51.5)
Regional	138(23.0)	400(22.6)	538(22.7)
Distant	31(5.2)	141(8.0)	172(7.2)
Unknown	136(22.6)	306(17.3)	442(18.6)

^a^Includes both melanoma (*n* = 184 for gestational group and *n* = 382 for postpartum group) and lip cancer (*n* = 3 for gestational group and *n* = 13 for postpartum group)

Tables 3 and 5 present the birth management and maternal outcomes for women included in the analysis. The odds of serious maternal complications (MMOI) were significantly higher in the gestational (AOR 5.07, 95% CI 3.72 – 6.90) and postpartum (AOR 1.55, 95% CI 1.16 – 2.09) cancer groups than in the no-PAC group. Transfusion of blood or coagulation factors was the single greatest contributor to the higher MMOI observed in the gestational group; 5.9% of women in this group required a blood transfusion or clotting factors compared to 1.7% in the postpartum group and 1.4% in the no-PAC group. Women diagnosed with cancer in pregnancy or postpartum were at significantly increased odds of induction or no-labor birth (AOR 2.10, 95% CI 1.78 – 2.47 and AOR 1.17, 95% CI 1.07 – 1.28, respectively) and CS (AOR 1.63, 95% CI 1.42 – 1.88 and AOR 1.14, 95% CI 1.06 – 1.24, respectively) compared to women in the no-PAC group. There were six maternal deaths within 42 days after birth among women with gestational cancer. Cancer was the cause of death in all these cases.

**Table 3:** Maternal outcomes of birthing women in NSW between 1994 and 2013, stratified by PAC status

Factors	Gestational	Postpartum	no-PAC	Total
	**N(%)**	**N(%)**	**N(%)**	**N(%)**
**Total**	601(100.0)	1772(100.0)	1,786,078 (100.0)	1,788,451(100.0)
**MMOI**^ab^				
Yes	46(11.3)	45(3.8)	26,444(2.4)	26,535(2.4)
No	362(88.7)	1139(96.2)	1,057,915(97.6)	1,059,416(97.6)
**Gestational diabetes**				
Yes	22(3.7)	101(5.7)	81,623(4.6)	81,746(4.6)
No	579(96.3)	1671(94.3)	1,704,455(95.4)	1,706,705(95.4)
**Gestational hypertension**				
Yes	38(6.3)	113(6.4)	109,051(6.1)	109,202(6.1)
No	563(93.7)	1659(93.6)	1,677,027(93.9)	1,679,249(93.9)
**Onset of labor**				
Spontaneous	233(38.8)	946(53.4)	1,088,985(61.0)	1,090,164(61.0)
Induced	187(31.1)	470(26.5)	438,995(24.6)	439,652(24.6)
No labor	181(30.1)	355(20.0)	257,643(14.4)	258,179(14.4)
Unknown	0(0.0)	1(0.1)	455(0.0)	456(0.0)
**Type of delivery**				
Vaginal	295(49.1)	1026(57.9)	1,140,733(63.9)	1,142,054(63.9)
Instrumental vaginal	50(8.3)	185(10.4)	191,746(10.7)	191,981(10.7)
Cesarean section	256(42.6)	560(31.6)	452,709(25.3)	453,525(25.4)
Unknown	0(0.0)	1(0.1)	890(0.0)	891(0.0)
**Gestational age at delivery**				
< = 28 weeks	11(1.8)	18(1.0)	13,380(0.7)	13,409(0.7)
29—31 weeks	27(4.5)	17(1.0)	8961(0.5)	9005(0.5)
32—36 weeks	113(18.8)	121(6.8)	93,260(5.2)	93,494(5.2)
> = 37 weeks	450(74.9)	1616(91.2)	1,670,236(93.5)	1,672,302(93.5)
Unknown	0(0.0)	0(0.0)	241(0.0)	241(0.0)
**Discharge status (transferred)**				
Yes	33(5.5)	67(3.8)	59,176(3.3)	59,276(3.3)
No	568(94.5)	1705(96.2)	1,726,902(96.7)	1,729,175(96.7)

^a^Maternal Morbidity Outcome Indicator (MMOI) occurring during pregnancy or within 42 days of giving birth [21]

^b^Data were available from 2001 Jul onwards

**Fig. 1:**
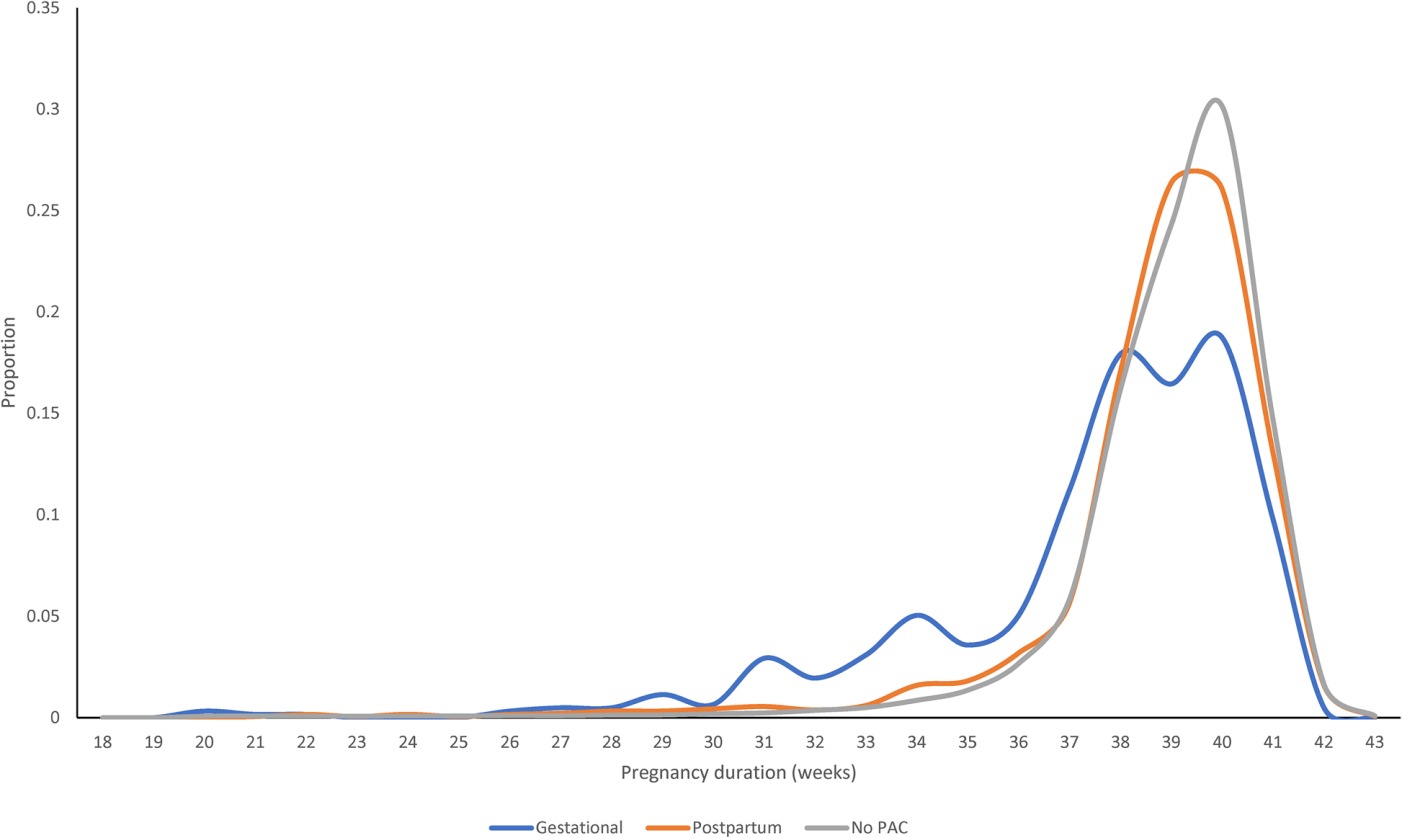
Pregnancy duration distribution among babies born in NSW between 1994 – 2013

Tables 4 and 5 present the perinatal outcomes of babies born to women in the study cohort. The mean gestational age at birth of babies born to women diagnosed with gestational cancer (37.4 ± 3.3 weeks) was lower than that of babies born to women with postpartum cancer (38.7 ± 2.4 weeks, mean difference 1.3 week, 95% CI 1.0 – 1.5, *p*-value < 0·001). Furthermore, the mean gestational age at birth of babies born to women in both these study groups was lower than that of babies born to women in the no-PAC group (39.0 ± 2.2 weeks; mean difference 1.5 weeks (95% CI 1.3 – 1.7) and 0.3 weeks (95% CI 0.2 – 0.4) respectively; *p*-value < 0·001 in both instances) (Fig. 1).

**Table 4:** Perinatal outcomes of babies born in NSW between 1994 and 2013

Factors	Gestational	Postpartum	no-PAC	Total
	**N(%)**	**N(%)**	**N(%)**	**N(%)**
**Total**	614(100.0)	1816(100.0)	1,813,292(100.0)	1,815,722(100.0)
**Perinatal death**				
Live > 28 days	608(99.0)	1799(99.1)	1,795,978(99.0)	1,798,385(99.0)
Perinatal death	6(1.0)	16(0.9)	17,059(0.9)	17,081(0.9)
Unknown	0(0)	1(0.1)	255(0.0)	256(0.0)
**Preterm**				
< 37 weeks	156(25.4)	184(10.1)	129,637(7.1)	129,977(7.2)
> = 37 weeks	458(74.6)	1632(89.9)	1,683,411(92.8)	1,685,501(92.8)
Unknown	0(0.0)	0(0.0)	244(0.0)	244(0.0)
**Planned preterm**^a^				
Yes	115(18.7)	74(4.1)	43,853(2.4)	44,042(2.4)
No	41(6.7)	110(6.1)	85,784(4.7)	85,935(4.7)
Not applicable	458(74.6)	1632(89.9)	1,683,655(92.9)	1,685,745(92.8)
**NAOI**^bc^				
Yes	74(16.9)	83(6.5)	60,062(5.2)	60,219(5.2)
No	365(83.1)	1189(93.5)	1,093,389(94.8)	1,094,943(94.8)
**Low birthweight**^c^				
< 2500 g	117(19.2)	142(7.9)	103,896(5.8)	104,155(5.8)
> = 2500 g	492(80.8)	1665(92.1)	1,697,220(94.2)	1,699,377(94.2)
Unknown	0(0.0)	0(0.0)	713(0.0)	713(0.0)
**SGA**^c^				
Yes	50(8.2)	183(10.1)	184,110(10.2)	184,343(10.2)
No	559(91.8)	1621(89.7)	1,614,179(89.6)	1,616,359(89.6)
Unknown	0(0.0)	3(0.2)	3540(0.2)	3543(0.2)
**LGA**^c^				
Yes	73(12.0)	218(12.1)	177,888(9.9)	178,179(9.9)
No	536(88.0)	1586(87.8)	1,620,401(89.9)	1,622,523(89.9)
Unknown	0(0.0)	3(0.2)	3540(0.2)	3543(0.2)
**Apgar at 5min**^c^				
0 to 3	4(0.7)	8(0.4)	5770(0.3)	5782(0.3)
4 to 6	18(3.0)	26(1.4)	23,050(1.3)	23,094(1.3)
7 to 10	587(96.4)	1767(97.8)	1,767,191(98.1)	1,769,545(98.1)
Unknown	0(0.0)	6(0.3)	5818(0.3)	5824(0.3)
**Admitted to ICU**^cd^				
Yes	194(31.9)	362(20.0)	296,258(16.4)	296,814(16.5)
No	415(68.1)	1445(80.0)	1,504,455(83.5)	1,506,315(83.5)
Unknown	0(0.0)	0(0.0)	1116(0.1)	1116(0.1)
**Length of stay**^ce^				
< 5 days	290(51.3)	1096(65.0)	1,185,806(69.3)	1,187,192(69.3)
5 or more	275(48.7)	589(34.9)	523,106(30.6)	523,970(30.6)
Unknown	0(0.0)	2(0.1)	1698(0.1)	1700(0.1)
**Congenital condition**^f^				
Yes	0(0.0)	5(1.5)	4765(1.6)	4770(1.6)
No	124(100.0)	339(98.5)	288,073(98.4)	288,536(98.4)

^a^Including labor induction and cesarean delivery without labor where main indications for cesarean section are not 'Failure to progress' or 'Fetal distress'

^b^Neonatal Adverse Outcome Indicator (NAOI) identified from a birth record or in any hospital transfer admission prior to the first discharge home [22]

^c^Live births only

^d^Admission to SCN or NICU for 4 h or more

^e^Only babies who were discharged home are included

^f^Register of Congenital Conditions are available for babies born in 2011—2013 only

**Table 5:** Multivariable analysis of maternal and perinatal outcomes for women with gestational and postpartum

Outcome^b^	Univariable analysis^a^		Multivariable analysis^a^	
	**Gestational**	**Postpartum**	**Gestational**^**c**^	**Postpartum**^**c**^
	**OR(95% CI)**	**OR(95% CI)**	**AOR(95% CI)**	**AOR(95% CI)**
Maternal outcomes				
MMOI^de^	5.11 (3.77 – 6.92)	1.60 (1.19 – 2.14)	5.07 (3.72 – 6.90)	1.55 (1.16 – 2.09)
Gestational diabetes	0.74 (0.48 – 1.14)	1.20 (0.99 – 1.46)	0.64 (0.41 – 1.00)	1.03 (0.85 – 1.26)
Gestational hypertension	0.98 (0.71 – 1.34)	1.02 (0.85 – 1.23)	0.91 (0.66 – 1.26)	1.02 (0.85 – 1.23)
Induced or no labor^f^	2.35 (2.01 – 2.75)	1.33 (1.22 – 1.45)	2.10 (1.78 – 2.47)	1.17 (1.07 – 1.28)
Cesarean section^g^	1.82 (1.59 – 2.09)	1.29 (1.20 – 1.40)	1.63 (1.42 – 1.88)	1.14 (1.06 – 1.24)
Discharge status (transferred)	1.72 (1.24 – 2.38)	1.11 (0.87 – 1.44)	1.94 (1.39 – 2.72)	1.17 (0.91 – 1.51)
Perinatal outcomes				
Perinatal death	0.94 (0.36 – 2.44)	0.89 (0.49 – 1.61)	0.90 (0.38 – 2.12)	0.85 (0.48 – 1.51)
Preterm	4.37 (3.58 – 5.34)	1.38 (1.16 – 1.64)	4.50 (3.63 – 5.58)	1.30 (1.09 – 1.54)
Planned preterm^h^	9.46 (7.63 – 11.73)	1.66 (1.28 – 2.16)	8.96 (6.96 – 11.53)	1.38 (1.05 – 1.81)
NAOI^i^	3.60 (2.79 – 4.64)	1.24 (0.98 – 1.57)	3.31 (2.52 – 4.35)	1.16 (0.92 – 1.47)
Low birthweight	3.88 (3.16 – 4.76)	1.33 (1.10 – 1.60)	4.13 (3.28 – 5.20)	1.28 (1.05 – 1.56)
SGA	0.84 (0.65 – 1.10)	1.00 (0.86 – 1.16)	0.91 (0.68 – 1.20)	1.10 (0.94 – 1.28)
LGA	1.24 (0.98 – 1.59)	1.24 (1.08 – 1.43)	1.15 (0.90 – 1.47)	1.11 (0.97 – 1.28)
Apgar at 5min^j^	2.28 (1.46 – 3.56)	1.18 (0.83 – 1.67)	2.23 (1.43 – 3.47)	1.16 (0.82 – 1.65)
Admit to ICU	2.33 (1.96 – 2.77)	1.24 (1.10 – 1.40)	2.38 (2.00 – 2.84)	1.20 (1.06 – 1.35)
Length of stay	2.00 (1.71 – 2.33)	1.10 (1.00 – 1.21)	1.87 (1.59 – 2.21)	1.06 (0.95 – 1.17)
Congenital conditions	NA	0.87 (0.31 – 2.47)	NA	0.84 (0.30 – 2.37)

^a^No-PAC is the reference group

^b^All outcomes are dichotomous in GEE models; ‘unknown’ category are combined with ‘no’ unless otherwise specified

^c^Adjusted for maternal age, country of birth, remoteness, parity, history of cesarean section, plurality, timing of antenatal care, smoking during pregnancy, place of birth, pre-existing diabetes, pre-existing hypertension, chronic kidney disease prior to pregnancy, and cardiovascular disease prior to pregnancy

^d^Maternal Morbidity Outcome Indicator (MMOI) occurring during pregnancy or within 42 days postpartum [21]

^e^Data were available from 2001 Jul onwards

^f^(induced + no labor) vs. (spontaneous + unknown)

^g^CS vs. (vaginal + instrumental vaginal + unknown)

^h^yes vs. (no + not applicable)

^i^Neonatal Adverse Outcome Indicator (NAOI) identified from a birth record or in any hospital transfer admission prior to the first discharge home [22]

^j^(0 -3 + 4–6) vs. (7 – 10 + Unknown)

There was no difference in perinatal mortality between babies born to women with gestational cancer (AOR 0.90, 95% CI 0.38 – 2.12) or postpartum cancer (AOR 0.85, 95% CI 0.48 – 1.51) and babies born to women in the no-PAC group. Nonetheless, babies of women with gestational or postpartum cancers were more likely to be born via planned delivery (AOR 8.96, 95% CI 6.96 – 11.53 and AOR 1.38, 95% CI 1.05 – 1.81, respectively). Babies born to women diagnosed with gestational cancer were at increased odds of a severe neonatal adverse outcome (NAOI) (AOR 3.31, 95% CI 2.52 – 4.35). This was not observed for babies born to women diagnosed with postpartum cancer (AOR 1.16, 95% CI 0.92 – 1.47).

## Discussion

Results from this study demonstrate an increase in PAC incidence in NSW over two decades that was independent of maternal age. Obstetric management of women with PAC, particularly those with gestational cancers, was associated with increased birth interventions, notably labor induction and planned CS. Women with gestational cancer, and to a lesser extent women with postpartum cancer, were at increased risk of serious maternal complications. Infants born to women with gestational cancer were more likely to be born preterm via labor induction or no-labor CS, be of low birthweight but not SGA, experience serious neonatal adverse outcomes, and be admitted to NICU/SCN than infants born to women without PAC. These findings suggest that the majority of babies of women with gestational cancer were born preterm for maternal or obstetric indications rather than for fetal reasons.

### Maternal complications

Women with PAC were at increased risk of a serious maternal morbidity outcome compared to women without PAC. We also found that women with gestational cancer were more likely to experience a serious maternal morbidity outcome than women with postpartum cancer, as has been previously reported [2]. It has previously been reported that a major contributor to increased maternal morbidities in women with gestational cancer is their increased risk of thrombosis [9]. However, the incidence of thrombotic events in our cohort was low, while the greatest contributor to severe maternal morbidity outcomes was blood transfusion or administration of clotting factor and, therefore, we cannot confirm this effect.

While women with postpartum cancer experienced fewer serious maternal morbidities than women with gestational cancer, this former group still exhibited significantly increased odds of serious maternal morbidities than women without PAC. One would expect that if no cancer was present in these women during their pregnancy, they should experience similar maternal outcomes to those without PAC. Hence, our findings may indicate that a subset of women in our postpartum group may in fact, have had cancer during pregnancy but remained undiagnosed, potentially due to masking of cancer signs and/or symptoms by physiological changes associated with pregnancy [3].

### Increased birth interventions

We found that women with PAC were more likely to have a planned delivery (labor induction or no-labor CS) than women without PAC, as reported in previous studies [2, 23]. While the higher rates of planned delivery in women with gestational cancer may be associated with decisions relating to cancer management, this cannot be the case for women diagnosed postpartum. In this latter group, it is possible that increased rates of planned delivery related to physiological impacts of undiagnosed cancer during pregnancy or coincidental chronic conditions.

There is a gap in the literature and current guidelines regarding the timing of planned CS for women with gestational cancer [24]. Optimizing the timing of delivery for women with gestational cancer requires a careful balancing between women's health outcomes, gestational age, and the newborn risks of early planned birth. A multidisciplinary team management approach, including clinicians from cancer and high-risk obstetrics disciplines, is necessary to ensure best possible outcomes for mothers and their babies [25].

Classic decision and risk theory would suggest that whilst decisions are often made without definitive knowledge of their consequences, decision making under uncertainty involves evaluation of the desirability of possible outcomes and the likelihood of their occurrence [26]. Obstetric clinical decision making in women with gestational cancer is influenced by considerable uncertainty. Hall argues that there are multiple sources of uncertainty for clinicians and most clinicians are either unaware of or will deny uncertainty [26]. In response to such uncertainty, clinicians may react in many ways including upholding medical orthodoxy, exhibiting considerable stress, or taking intuitive action, such as planned early birth [26]. This latter response may partially explain the high rate of planned early birth we observed in our gestational cancer cohort. Nonetheless, cancer outcomes are generally improved with early intervention [27, 28] and hence this finding may reflect decisions by treating oncologists and obstetricians to balance the conflicting health needs of the mother with those of their babies (i.e., to treat the mother as soon as possible to improve cancer outcomes while delaying the birth until as late as possible to improve baby outcomes).

### Perinatal outcomes for infants

We report that the odds of perinatal death among the PAC groups and the no-PAC group were not significantly different. These results differ from some previously published studies that reported significantly higher stillbirth rates and neonatal death among babies born to women with PAC [29]. The high rate of planned delivery by CS in our study groups may be associated with the observed lower perinatal death rate, as planned CS delivery can reduce the incidence of intrapartum stillbirth [30]. Alternatively, this finding may relate to the high quality of health care available in Australia which may increase the chances of survival of babies born to women with gestational cancer. Note that the availability of high quality health care in Australia may also explain why there was no increase in the odds of perinatal mortality among babies born to women with gestational cancer despite these babies having increased odds of adverse severe neonatal outcomes compared to babies born to women in the no-PAC group.

Our results confirmed findings from previous studies that infants born to women with PAC are more likely to be born preterm and to be of low birthweight but that there is no significant association between PAC and the odds of a baby being born SGA [5]. These results suggest that the high prevalence of low birthweight observed in PAC infants is more likely to be due to preterm birth rather than restricted fetal growth. In addition, the comparable prevalence of SGA observed in infants born to women with gestational cancer and women without PAC may reflect the increased frequency of antenatal visits and antenatal monitoring among women with gestational cancer. This suggestion is supported by our finding that a higher proportion of women with gestational cancer in our cohort commenced their antenatal care before the 14^th^ week of gestation compared to women without PAC.

We found that infants born to women with gestational cancer are more likely to experience a severe adverse neonatal outcome than infants born to women without PAC. This finding is likely to be a consequence of the higher prevalence of preterm birth among infants in the gestational group. Lain and colleagues previously reported that preterm infants have a higher incidence of adverse neonatal outcomes than term infants [19] and that, in the context of planned birth, every additional week of gestation towards the end of pregnancy mitigates against potential adverse neonatal outcomes [31]. Therefore, guidance on timing of birth is critical in minimizing risk to the neonate.

### Incidence

We estimated crude PAC incidence in NSW as 132/100,000 women giving birth. In agreement with previously reported studies, we found that PAC incidence increased over the 20-year study period [2, 32]. Even when adjusting for age, this increase remained significant, suggesting that other factors may be associated with this trend. It is noteworthy that melanoma was the major contributor to diagnoses of PAC in our study cohort. This contrasts previous international studies that report breast cancer as the major contributor to PAC [1, 5, 8] and reflects the overall high incidence of skin cancers in Australia [33].

### Potential delay in diagnosis

It has been suggested that estimates of gestational cancer incidence are likely to be lower than the actual incidence of disease owing to potential delayed diagnosis of cancers present during pregnancy [34]. Our results are consistent with this hypothesis as cases of gestational cancer represented only one-quarter of all PAC diagnoses. Given that the at-risk period for a diagnosis of cancer in the postpartum group is only approximately 27% longer than in the gestational group (52 weeks vs. up to ~ 41 weeks for a full-term birth), we would expect that postpartum cancer incidence would be approximately 1.27 times higher than gestational cancer incidence. Our finding that postpartum cancer incidence was 2.9 times higher than gestational cancer strongly suggests that some cases of postpartum cancer were undiagnosed gestational cancers.

Several factors may be associated with the lower rate of cancer diagnosis during pregnancy. The initial diagnosis of cancer during pregnancy can be challenging as signs and symptoms of cancer can be incorrectly attributed to the pregnancy [3]. Furthermore, there is limited evidence defining reference ranges in pregnant women for laboratory markers relevant to the recognition, diagnosis, and prognostication of cancers [35]. Finally, physicians may be hesitant to request investigations (e.g., imaging investigations) that may potentially impact the fetus [36–38].

### Strengths and limitations

This study is truly population-based because, in NSW, there is mandatory notification of all cancer diagnoses to the cancer registry and maternity datasets cover all sources (public and private hospitals, and the community). The main limitation of this study is that the dataset lacks information on early pregnancy loss, restricting the study analyses to pregnancies that resulted in a birth. Furthermore, a lack of information on cancer treatment limited our ability to examine outcomes by type and timing of treatment. These limitations highlight the need for comprehensive data collection through national data assets and international registries with high-quality data to enable clinicians and patients to make better informed decisions surrounding clinical management. It should also be noted that the postpartum group was defined using an exposure that occurred after the outcome measures. Hence, caution is required when interpreting results for this group.

## Conclusion

PAC incidence in NSW is increasing, and this increase appears to be independent of maternal age. Women with PAC are more likely to have serious maternal morbidities and their babies experience poorer perinatal outcomes associated with preterm birth. The higher rate of birth intervention among women with gestational cancers reflects the complexity of clinical decision making in providing a balanced outcome for women and their infants. The lower observed incidence of diagnoses of gestational cancer compared to postpartum cancer suggests a need to strengthen clinical surveillance and diagnostics to distinguish pregnancy-related symptoms from cancer symptoms to improve clinical practice. Future research is required investigating the prognosis for women with gestational cancer compared to age-matched women diagnosed with cancer outside of pregnancy or the postpartum period.

## Supplementary Information

**Additional file 1:** **Supplemental Table 1.** The population datasets used in this study. **Supplemental Table2.** The components of severe maternal morbidity outcome indicator (MMOI). **Supplemental Table 3.** The components of severe neonatal adverse outcome indicator (NAOI). **Supplemental figure 1.** Crude and age-standardized incidence of pregnancy-associated cancer in NSW (1994 – 2013).

## Data Availability

Primary data for this study comprised confidential health data that were provided by the New South Wales Ministry of Health, Australia. Owing to privacy regulations in force in Australia, these data cannot be shared publicly. It was a condition of the ethics approval obtained for this study that only those researchers specifically named in the ethics submission be able to access the deidentified linked data derived from the New South Wales Ministry of Health primary health datasets. The data for this study can be requested (with permission of the relevant ethics committee) from the Centre for Health Record Linkage (CHeReL).; email: MOH-CHeReL@health.nsw.gov.au.

## Abbreviations

AOR: Adjusted Odds Ratio
CS: Cesarean Section
MMOI: Severe Maternal Morbidity Outcome Indicator
NAOI: Severe Neonatal Adverse Outcome Indicator
NICU: Neonatal Intensive Care Unit
OR: Odds Ratio
PAC: Pregnancy-Associated Cancer
SCN: Special Care Nursery

## References

[1] Cottreau CM, Dashevsky I, Andrade SE, Li DK, Nekhlyudov L, Raebel MA (2019). Pregnancy-Associated Cancer: A U.S. Population-Based Study. J Womens Health (Larchmt).

[2] Lee YY, Roberts CL, Dobbins T, Stavrou E, Black K, Morris J (2012). Incidence and outcomes of pregnancy-associated cancer in Australia, 1994–2008: a population-based linkage study. BJOG.

[3] Smith LH, Dalrymple JL, Leiserowitz GS, Danielsen B, Gilbert WM (2001). Obstetrical deliveries associated with maternal malignancy in California, 1992 through 1997. Am J Obstet Gynecol.

[4] Dalmartello M, Negri E, La Vecchia C, Scarfone G, Buonomo B, Peccatori FA (2020). Frequency of Pregnancy-Associated Cancer: A Systematic Review of Population-Based Studies. Cancers (Basel).

[5] Esposito G, Franchi M, Dalmartello M, Scarfone G, Negri E, Parazzini F (2021). Obstetric and neonatal outcomes in women with pregnancy associated cancer: a population-based study in Lombardy, Northern Italy. BMC Pregnancy Childbirth.

[6] Momen NC, Arendt LH, Ernst A, Olsen J, Li J, Gissler M (2018). Pregnancy-associated cancers and birth outcomes in children: a Danish and Swedish population-based register study. BMJ Open.

[7] Safi N, Saunders C, Hayen A, Anazodo A, Lui K, Li Z (2021). Gestational breast cancer in New South Wales: A population-based linkage study of incidence, management, and outcomes. PLoS ONE.

[8] Smith LH, Danielsen B, Allen ME, Cress R (2003). Cancer associated with obstetric delivery: results of linkage with the California cancer registry. Am J Obstet Gynecol.

[9] Greiber I, Mikkelsen A, Karlsen M, Storgaard L, Viuff J, Mellemkjær L (2021). Cancer in pregnancy increases the risk of venous thromboembolism: a nationwide cohort study. BJOG.

[10] Amant F, Han SN, Gziri MM, Vandenbroucke T, Verheecke M, Van Calsteren K (2015). Management of cancer in pregnancy. Best Pract Res Clin Obstet Gynaecol.

[11] Safi N, Anazodo A, Dickinson JE, Lui K, Wang AY, Li Z (2019). In utero exposure to breast cancer treatment: a population-based perinatal outcome study. Br J Cancer.

[12] Centre for Epidemiology and Evidence (2021). New South Wales Mothers and Babies 2019.

[13] Centre for Health Record Linkage (CHeReL). Data dictionaries. CHeReL. 2022. https://www.cherel.org.au/data-dictionaries. Accessed 30 Sept 2022.

[14] Cancer Institute NSW. NSW Cancer Registry – Data dictionary - NSW. Cancer Institute NSW. https://www.cancer.nsw.gov.au/getmedia/c86d5384-d898-47e3-88f6-ce7edaef67e7/E21-02235-NSWCR-datadictionary-2018-preliminary-CIM-release.pdf. Accessed 5 Oct 2022.

[15] Centre for Health Record Linkage (CHeReL). How record linkage works. Centre of Health Record Linkage (CHeReL). https://www.cherel.org.au/how-record-linkage-works. Accessed 30 Sept 2022.

[16] Roberts CL, Cameron CA, Bell JC, Algert CS, Morris JM (2008). Measuring maternal morbidity in routinely collected health data: development and validation of a maternal morbidity outcome indicator. Med Care.

[17] Dobbins TA, Sullivan EA, Roberts CL, Simpson JM (2012). Australian national birthweight percentiles by sex and gestational age, 1998–2007. Med J Aust.

[18] Li Z, Umstad MP, Hilder L, Xu F, Sullivan EA (2015). Australian national birthweight percentiles by sex and gestational age for twins, 2001–2010. BMC Pediatri.

[19] Lain SJ, Algert CS, Nassar N, Bowen JR, Roberts CL (2012). Incidence of severe adverse neonatal outcomes: use of a composite indicator in a population cohort. Matern Child Health J.

[20] R Core Team (2020). R: A language and environment for statistical computing.

[21] Roberts CL, Cameron CA, Bell JC, Algert CS, Morris JM (2008). Measuring maternal morbidity in routinely collected health data: development and validation of a maternal morbidity outcome indicator. Med Care.

[22] Lain SJ, Algert CS, Nassar N, Bowen JR, Roberts CL (2012). Incidence of severe adverse neonatal outcomes: use of a composite indicator in a population cohort. Matern Child Health J.

[23] Van Calsteren K, Heyns L, De Smet F, Van Eycken L, Gziri MM, Van Gemert W (2010). Cancer during pregnancy: an analysis of 215 patients emphasizing the obstetrical and the neonatal outcomes. J Clin Oncol.

[24] Coates D, Homer C, Wilson A, Deady L, Mason E, Foureur M (2020). Indications for, and timing of, planned caesarean section: A systematic analysis of clinical guidelines. Women and Birth.

[25] Zagouri F, Dimitrakakis C, Marinopoulos S, Tsigginou A, Dimopoulos MA (2016). Cancer in pregnancy: disentangling treatment modalities. ESMO Open.

[26] Hall KH (2002). Reviewing intuitive decision-making and uncertainty: the implications for medical education. Med Educ.

[27] Brkljačić B, Šupe PA (2020). Croatian success in early breast cancer detection: favorable news in Breast Cancer Awareness Month. Croat Med J.

[28] Neal RD, Tharmanathan P, France B, Din NU, Cotton S, Fallon-Ferguson J (2015). Is increased time to diagnosis and treatment in symptomatic cancer associated with poorer outcomes? Systematic review. Br J Cancer.

[29] Lu D, Ludvigsson JF, Smedby KE, Fall K, Valdimarsdóttir U, Cnattingius S (2017). Maternal Cancer During Pregnancy and Risks of Stillbirth and Infant Mortality. J Clin Oncol.

[30] Darmstadt GL, Yakoob MY, Haws RA, Menezes EV, Soomro T, Bhutta ZA (2009). Reducing stillbirths: interventions during labour. BMC Pregnancy Childbirth.

[31] Australian Commission on Safety and Quality in Health Care and Australian Institute of Health and Welfare. Chapter 1: Early planned births without medical or obstetric indication. In: The Fourth Australian Atlas of Healthcare Variation. Sydney: ACSQHC; 2021. Available from: https://www.safetyandquality.gov.au/sites/default/files/2021-04/fourth_atlas_2021_-_1.1_early_planned_birth_without_medical_or_obstetric_indication.pdf.

[32] Eibye S, Kjaer SK, Mellemkjaer L (2013). Incidence of pregnancy-associated cancer in Denmark, 1977–2006. Obstet Gynecol.

[33] Sung H, Ferlay J, Siegel RL, Laversanne M, Soerjomataram I, Jemal A (2021). Global Cancer Statistics 2020: GLOBOCAN Estimates of Incidence and Mortality Worldwide for 36 Cancers in 185 Countries. CA: Cancer J Clin.

[34] Andersson TM, Johansson AL, Fredriksson I, Lambe M (2015). Cancer during pregnancy and the postpartum period: A population-based study. Cancer.

[35] Di Ciaccio PR, Mills G, Tang C, Hamad N (2021). Managing haematological malignancies in pregnant women. Lancet Haematol.

[36] Amant F, Loibl S, Neven P, Van Calsteren K (2012). Breast cancer in pregnancy. Lancet.

[37] Safi N, Saunders C, Anazodo A, Dickinson JE, Boyle F, Ives A, et al. Clinical decision making in the management of breast cancer diagnosed during pregnancy: a review and case series analysis. J Adolesc Young Adult Oncol. 2022;11(3):245–51. 10.1089/jayao.2021.0054 34813371

[38] Di Ciaccio PR, Emmett L, Hamad N (2021). Qualitative study of nuclear medicine physicians' perceptions of positron emission tomography/computed tomography in pregnant patients with cancer. Intern Med J.

