# Supplementary material for: Pregnancy associated cancer, timing of birth and clinical decision making—a NSW data linkage study
Source: BMC Pregnancy Childbirth. 2023 Feb 9;23:105. doi: 10.1186/s12884-023-05359-1 (PMC9909861; doi:10.1186/s12884-023-05359-1)
Supplement: Supplementary file 1 — Additional file 1: Supplemental Table 1. The population datasets used in this study. Supplemental Table2. The components of severe maternal morbidity outcome indicator (MMOI). Supplemental Table 3. The components of severe neonatal adverse outcome indicator (NAOI). Supplemental figure 1. Crude and age-standardized incidence of pregnancy-associated cancer in NSW (1994 – 2013). [file 12884_2023_5359_MOESM1_ESM.docx]

**Additional material**

**Table of content:**

1. Supplemental Table 1: The population datasets used in this study.
2. Supplemental Table2: The components of severe maternal morbidity outcome indicator (MMOI).
3. Supplemental Table 3: The components of severe neonatal adverse outcome indicator (NAOI).
4. Supplemental figure 1: Crude and age-standardized incidence of pregnancy-associated cancer in NSW (1994 – 2013).

**Supplemental Table 1: Population datasets used in this study**

| 1 | NSW Perinatal Data Collection (PDC) |
| --- | --- |
| 2 | NSW Cancer Registry (NSWCR) |
| 3 | NSW Admitted Patient Data Collection (APDC) |
| 4 | Register of Congenital Conditions (RoCC) |
| 5 | Registrar of Births, Deaths and Marriages (RBDM) |
| 6 | Cause of Death Unit Record File (COD URF) |
| 7 | Perinatal Death Review (PDR) |

**Supplemental table 2: The components of severe maternal morbidity outcome indicator (MMOI)**

| **Morbidities** | **Procedures** |
| --- | --- |
| Acute abdomen | Assisted ventilation |
| Acute renal failure | Curettage in combination with general anaesthetic |
| Acute psychosis | Dialysis |
| Cardiac arrest / failure / infarction | Evacuation of hematoma |
| Cerebral oedema or coma | Hysterectomy |
| Disseminated intravascular coagulopathy | Procedures to reduce blood flow to uterus |
| Cerebrovascular accident | Reclosure of disrupted CS wound |
| Major complications of anaesthesia | Repair of bladder or cystostomy |
| Obstetric embolism | Repair of intestine |
| Shock | Repair ruptured or inverted uterus |
| Sickle cell anaemia with crisis | Transfusion of blood or coagulation factors |
| Status asthmaticus |  |
| Status epilepticus |  |
| Uterine rupture |  |

**Supplemental table 3: The components of severe neonatal adverse outcome indicator (NAOI)**

| **Diagnosis** | **Procedures** |
| --- | --- |
| Respiratory distress syndrome, other respiratory: primary atelectasis, respiratory failure | Resuscitation |
| Seizure | Ventilatory support (mechanical ventilation and/or CPAP) |
| Intraventricular haemorrhage | Central venous or arterial catheter |
| Cerebral infarction | Transfusion of blood or blood products |
| Periventricular leukomalacia | Pneumothorax requiring an intercostal catheter |
| Birth trauma | Any body cavity surgical procedure |
| Hypoxic ischemic encephalopathy | Any intravenous fluids |
| Necrotizing enterocolitis |  |
| Broncho-pulmonary dysplasia |  |
| Sepsis/septicaemia |  |
| Pneumonia |  |
| other respiratory conditions: primary atelectasis, respiratory failure |  |

**Supplemental Figure 1** Crude and age-standardized incidence of pregnancy-associated cancer in NSW (1994 – 2013).
